# Preparation of Cu-MnO_2_/GO/PVDF Catalytic Membranes via Phase Inversion Method and Application for Separation Removal of Dyes

**DOI:** 10.3390/membranes15120384

**Published:** 2025-12-18

**Authors:** Fei Wang, Xinyu Hou, Runze He, Jiachen Song, Yifan Xie, Zhaohui Yang, Xiao Liu

**Affiliations:** 1 School of Materials Science & Engineering, North Minzu University, Yinchuan 750021, China; wf20021030@163.com (F.W.); 16604696228@163.com (X.H.); 15591779243@163.com (R.H.); songjiachen2004@163.com (J.S.); 16636190089@163.com (Y.X.); 15198771891@163.com (Z.Y.); 2 Institute of Functional and Structural Characteristic Materials, Helanshan Laboratory, Yinchuan 750021, China

**Keywords:** membrane separation technique, advanced oxidation technology, Cu-MnO_2_, PVDF

## Abstract

To address the issues of hydrophobicity, easy fouling, and limited application of polyvinylidene fluoride (PVDF) membranes in water treatment processes, this study prepared Cu-MnO_2_/GO/PVDF catalytic membranes via the immersion precipitation phase inversion method. Graphene oxide (GO) was incorporated to facilitate the construction of good water channels, while copper-doped manganese dioxide (Cu-MnO_2_) was added to enhance catalytic activity. The structure, morphology, and performance of the membranes were characterized comprehensively. Results showed that Cu-MnO_2_ was well interspersed between GO sheets, thereby increasing membrane surface roughness, effective filtration area, and hydrophilicity. The best catalytic membrane CM-5 exhibited the highest pure water flux (1391.20 L·m^−2^·h^−1^) and methyl blue (MBE) rejection rate (98.06%), and it also displayed excellent reusability and stability. EPR tests confirmed the generation of HO· and HOO· in the Fenton-like system, which mediated dye degradation. The Cu-MnO_2_/GO/PVDF catalytic membrane demonstrated excellent hydrophilicity, antifouling properties, and catalytic efficiency, thus providing a viable solution for dye wastewater treatment.

## 1. Introduction

Polyvinylidene fluoride (PVDF) is one of the most commonly used membrane materials, due to its high mechanical strength, thermal stability [1], good chemical corrosion resistance, high temperature resistance, oxidation resistance, climate resistance, radiation resistance, piezoelectric, dielectric, thermoelectric, and other special properties. It has been proven to be versatile in membrane science and is also the most widely used material for fabricating polymer filter membranes in recent years [2,3,4,5]. At present, the commonly used preparation method is the immersion precipitation phase inversion method. By adjusting the composition of the casting solution, coagulation bath, and coagulation temperature, various catalytic membranes with distinct catalytic and retention performances can be prepared. Some studies have reported the fabrication of PVDF composite membranes by the phase inversion method [6,7]. However, PVDF is a hydrophobic material [8], and the prepared catalytic membrane exhibits a large resistance in the water treatment process and is easily fouled, which diminishes the performance of the membrane. Thus, the hydrophobicity of PVDF membrane limits its application in the water treatment industry [9]. Therefore, it is very necessary to modify the PVDF catalytic membrane, with the aim of enhancing the hydrophilicity, rejection capacity, and antifouling properties of the catalytic membrane.

Manganese has many oxide forms, such as MnO, MnO_2_, Mn_3_O_2_, Mn_3_O_4_, Mn_5_O_8_, and MnOOH. Through research and comparison, the catalytic activity is ranked as follows: Mn_5_O_8_ < Mn_3_O_4_ < Mn_3_O_2_ < MnO < MnOOH < MnO_2_ [10]. Therefore, manganese dioxide (MnO_2_) has high chemical activity as a catalyst; moreover, it is widespread in nature, low in price, and non-toxic. In this paper, copper-doped manganese dioxide is used as a catalyst to further enhance the catalytic activity of the membrane. Therefore, the membrane material prepared by combining this high activity catalyst with the phase inversion method can not only enhance the interfacial adsorption of organic pollutants via the developed pores of Cu-MnO_2_, but also improve the catalytic activity and expand the application pH range of Fenton-like advanced oxidation technology.

In recent years, graphene oxide (GO) has attracted wide attention due to its fascinating mechanical, electrical, thermal, and optical properties. Compared with other carbon materials, GO has perfect sp^2^ hybrid carbon nanostructures and a variety of oxygen groups, including epoxy groups, hydroxyl groups, carbonyl groups, and carboxyl groups, which can be easily dispersed in organic solvents via ultrasound effect to form a single GO nanosheet [11,12]. In addition, the incorporation of GO into PVDF membranes plays a crucial role in optimizing membrane performance from multiple dimensions. On one hand, GO is rich in oxygen-containing functional groups on its surface, which can significantly enhance the hydrophilicity of PVDF membranes and effectively mitigate the intrinsic hydrophobicity of PVDF, a property that often leads to high water transport resistance and easy fouling in water treatment processes [12,13]. On the other hand, GO exhibits a unique two-dimensional lamellar structure, which not only provides a stable “framework” for the PVDF membrane matrix but also could create favorable conditions for the intercalation of nanocatalysts. When nanocatalysts are intercalated between GO lamellae, a novel hierarchical water channel structure can be constructed within the PVDF membrane; this structure could allow small water molecules to pass through smoothly while exerting effective physical interception on macromolecular organic pollutants simultaneously [14,15,16].

Copper-doped manganese dioxide (Cu-MnO_2_) is a novel catalyst developed by our own laboratory, which has excellent catalytic performance. Notably, research about the combined modification of PVDF using Cu-MnO_2_ and GO remains scarce [17]. Innovatively, this study combines Cu-MnO_2_ with GO, enabling Cu-MnO_2_ to be uniformly interspersed between GO sheets. This design not only leverages the oxygen-containing functional groups and lamellar structure of GO to construct high-performance water channels but also relies on Cu doping to enhance the catalytic activity of MnO_2_, whereby the functional complementarity of “hydrophilic channel construction” and “catalytic activity enhancement” can be achieved.

Herein, Cu-MnO_2_/GO/PVDF catalytic membranes with different components are prepared by the immersion precipitation phase inversion method, and different characterizations are used to study the effect of GO and Cu-MnO_2_ amount on the performance of catalytic membranes. To evaluate the catalytic separation performance of the membranes, these membranes are used for the separation and purification of different organic wastewater, and the treatment effects on various organic wastewater are studied. Finally, the filtration mechanism of the membrane is analyzed and discussed.

## 2. Experiment

### 2.1. Materials

All the chemical reagents in this experiment belong to the analytical grade and did not need further purification. Potassium permanganate (KMnO_4_) and acetic acid were purchased from Shanghai Wokai Biotechnology Co., Ltd (Shanghai, China). Copper sulfate (CuSO_4_·5H_2_O) and sodium nitrate (NaNO_3_) were purchased from Shanghai Guangnuo Chemical Technology Co., Ltd. (Shanghai, China), ammonia was purchased from Tianjin Guangfu Technology Development Co., Ltd. (Tianjin, China), PVDF powder was purchased from France Arkema, and N-methylpyrrolidone (NMP) was purchased from Sinopharm Chemical Reagent Co., Ltd. (Tianjin, China). Graphite powder, concentrated sulfuric acid, concentrated hydrochloric acid, acetic acid, anhydrous ethanol, citric acid, disodium hydrogen phosphate, hydrogen peroxide, methyl blue (MBE), Congo red (CR), rhodamine b (Rh B), and methylene blue (MB) were purchased from Sinopharm Chemical Reagent Co., Ltd. (Tianjin, China). The above reagents are analytical-grade reagents, and the solvent of all solutions in the experiment was deionized water.

### 2.2. Preparation of GO and Cu-MnO_2_

GO was prepared using an improved Hummers method [18]. Natural flake graphite was oxidized in the mixture of H_2_SO_4_, NaNO_3_, and KMnO_4_. After the oxidation reaction completed, the product was obtained via the filtration of the solution and washed with deionized water. After drying in the oven at 50 °C for 48 h, GO powder was obtained by grinding. The Cu-MnO_2_ catalyst was prepared by the redox reaction between potassium permanganate and acetic acid, as we reported earlier [17]. Firstly, 0.6 g of KMnO_4_ was accurately weighed and dissolved in 30 mL 0.4 M acetic acid, and 0.3 g of CuSO_4_·5H_2_O was fully dissolved in 10 mL of distilled water. Secondly, the two solutions were fully mixed and transferred to a Teflon-lined autoclave with the addition of 40 µL of NH_3_·H_2_O. Then, the reaction was taken out at 140 °C for 12 h, and a dark brown precipitate (Cu-MnO_2_) was obtained after being dried in an oven at 60 °C for 12 h.

### 2.3. Preparation of Catalytic Membrane

**Figure 1:**
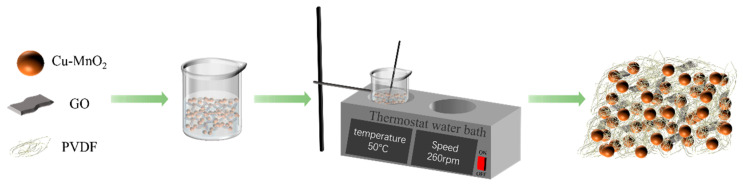
Preparation of Cu-MnO_2_ /GO/PVDF catalytic membrane.

To prepare catalytic membranes, 0.5 g of GO powder was added to a 100 mL beaker, followed by the addition of a certain amount of Cu-MnO_2_ catalyst. Then, 30 mL of NMP was added to the beaker, and the mixture was ultrasonically dispersed for 2 h and continuously stirred to obtain a homogeneous dispersion. Then, 3 g of PVDF powder was added to the homogeneous dispersion solution, and the mixture was placed in a 50 °C water bath with stirring at 260 r/min for 15 h. Next, the uniform casting solution was spread evenly onto the glass plate using a scraper, and the glass plate was placed in a water bath. After the phase transformation process, the separation membrane was obtained (Figure 1). The compositions of membranes with different compositions are listed in Table 1, denoted as CM-1, CM-2, CM-3, CM-4, and CM-5.

### 2.4. Instruments and Characteristics

The morphology and element distribution of membranes were studied using scanning electron microscopy (SEM, SIGMA 300, Oberkochen, Germany). The surface roughness of the membranes was measured by atomic force microscopy (AFM, Oxford MFP-3D infinity, Oxford, UK). The chemical structure of the catalytic membranes was analyzed using Fourier transform infrared spectrophotometer (FT-IR, Model 170-SX, Nicolet Instrument Corporation, Madison, WI, USA). X-ray diffraction (XRD, SmartlabSE, Tokyo, Japan) analysis was used to characterize the crystal structure of the membranes. X-ray photoelectron spectroscopy (XPS, Thermo Fisher Scientific, Waltham, MA, USA) was used to analyze the surface elemental composition of the catalytic membrane. The water contact angle (CA) was measured using a contact angle tester (JC2000D2, Shanghai, China). The charge of the catalytic membrane mixture was analyzed by a Malvern nanoparticle size and zeta potential analyzer (ZS90, Malvern Panalytical, Malvern, UK). The thermal stability of the membrane was tested using a thermogravimetric analyzer (TG, TG 309 Libra Supreme, Freiburg, German). Electron paramagnetic resonance (EPR, HJIN 46438, Augsburg, Germany) was used to prove the presence of free radicals in the system.

### 2.5. Average Contact Angle and Water Flux

CA and pure water flux (J) were measured to characterize the hydrophilicity and permeability of the prepared membranes. The data and images of the CA measurement were obtained after deionized water droplets were placed on the membrane surface at room temperature. The average contact angle was calculated by testing three distinct positions on each catalytic membrane. Prior to water flux determination, the membrane was pre-pressurized with deionized water until the water permeation reached a stable value (~0.1 MPa). The pure water flux (J) was tested via vacuum filtration and calculated according to the following Formula (1) [19]:

J = V/(A.t) (1)

Herein, V, A, and t represent the volume of deionized water (L), effective filtration area (m^2^), and operation time (h).

### 2.6. Separation and Degradation Evaluation

The separation performance of the catalytic membrane was evaluated using simulated dyes wastewater (MB, Rh B, CR, and MBE solutions). The filtration process was conducted under vacuum conditions. Prior to the experiment, a stable pressure (0.1 MPa) was established by pre-filtering deionized water. Then, 50 mL of simulated wastewater was filtered through the catalytic membrane, and the permeation flux of the contaminated solution as well as the rejection rate of various solutes were calculated. A UV spectrophotometer was used to test the concentration of dyes in the solution before and after filtration. The permeation flux (J) of simulated wastewater was calculated using Equation (2), and the retention rate (R) was defined as follows [19]:

R (%) = (1 − Cp/Cf) × 100%(2)

Herein, Cp and Cf represent the concentration of pollutants (mg/L) after permeation solution and feed solution, respectively.

## 3. Results and Discussion

### 3.1. Structure and Morphology Characterization of Membrane

**Figure 2:**
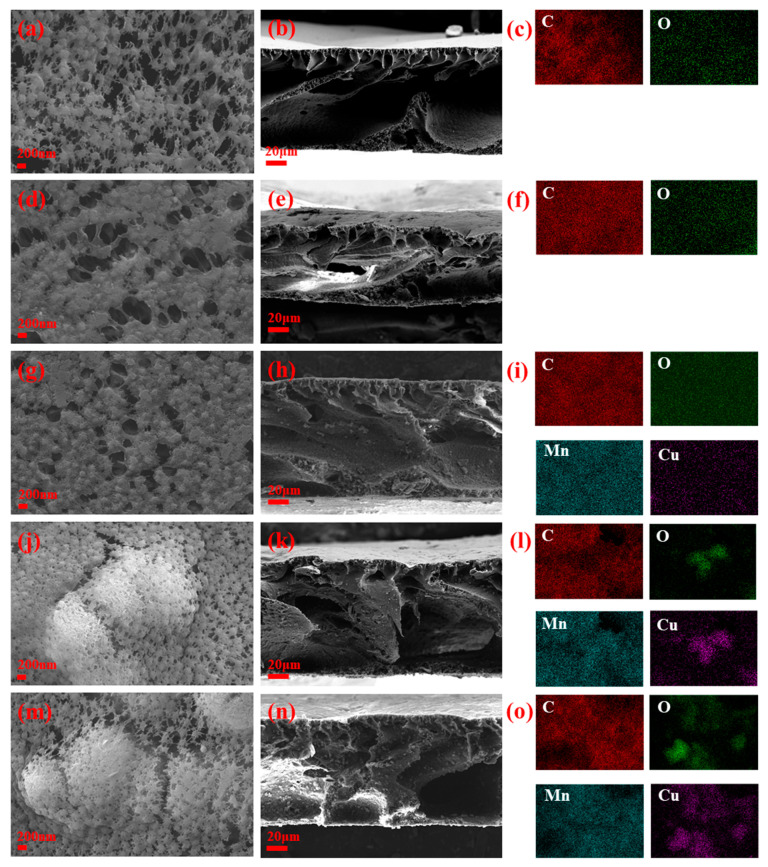
SEM images of the surface and cross-section, and EDS spectra of membranes: CM-1 (**a**–**c**), CM-2 (**d**–**f**), CM-3 (**g**–**i**), CM-4 (**j**–**l**), and CM-5 (**m**–**o**).

The surface and cross-section structure of the catalytic membrane was observed by SEM, as shown in Figure 2. It can be seen that the surface of CM-1 has numerous micropores with non-uniform sizes. With the addition of GO, CM-2 also exhibited many irregular pore structures. However, compared to CM-1, the surface structure of CM-2 was more compact, which was attributed to the formation of GO layer stacking on its surface. When Cu-MnO_2_ was incorporated (CM-3 to CM-5), the SEM images revealed a gradual change in both pore characteristics and particle distribution. For CM-3, Cu-MnO_2_ nanoparticles were observed to intersperse within the GO-modified PVDF matrix. The particles were relatively dispersed, and the pore structure remained well maintained, suggesting initial synergy between GO and Cu-MnO_2_ in membrane structure regulation. As the Cu-MnO_2_ loading increased, the nanoparticles became more prominent, which would lead to a decrease in the surface porosity of the membranes. However, the overall porous framework was preserved, even at the highest loading. From the cross-sectional images of the membranes, the thickness of the membranes was approximately 100 μm. Moreover, as the membrane composition changed, the cross-sectional structures also underwent significant alterations. On the cross-sections of CM-3, CM-4, and CM-5, obvious catalyst nanoparticles could be observed, indicating that the catalyst has been well dispersed and integrated with the interior of the membranes. Furthermore, the EDS results also confirmed the successful preparation of the composite membranes.

**Figure 3:**
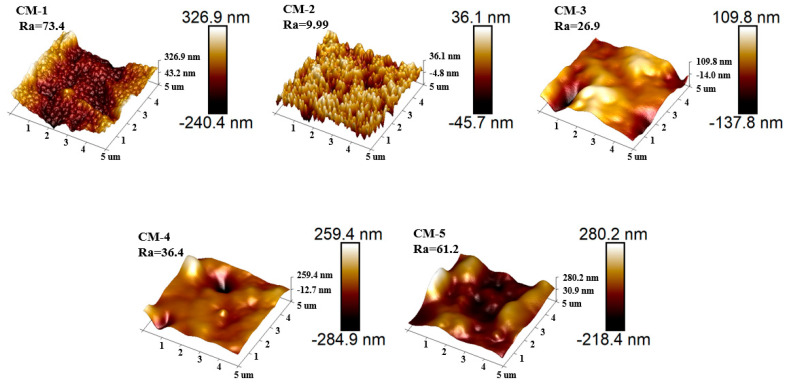
Surface AFM images of CM-1, CM-2, CM-3, CM-4, and CM-5.

The surface roughness of the membrane was characterized using AFM (Figure 3). As illustrated in the figure, the arithmetic mean roughness (Ra) of CM-1 was 73.4 nm. After the incorporation of GO, the roughness of CM-2 decreased drastically to 9.99 nm. However, with the sequential loading of the Cu-MnO_2_ catalyst, the roughness values of CM-3, CM-4, and CM-5 increased to 26.9 nm, 36.4 nm, and 61.2 nm, respectively, exhibiting a gradual upward trend with increasing Cu-MnO_2_ loading content. The presence of Cu-MnO_2_ on the membrane surface directly influenced its roughness, and the higher roughness values would corresponded to a larger effective filtration area, which could not only facilitate the exposure of active catalytic sites but also promotes mass transfer during the filtration–catalysis process.

**Figure 4:**
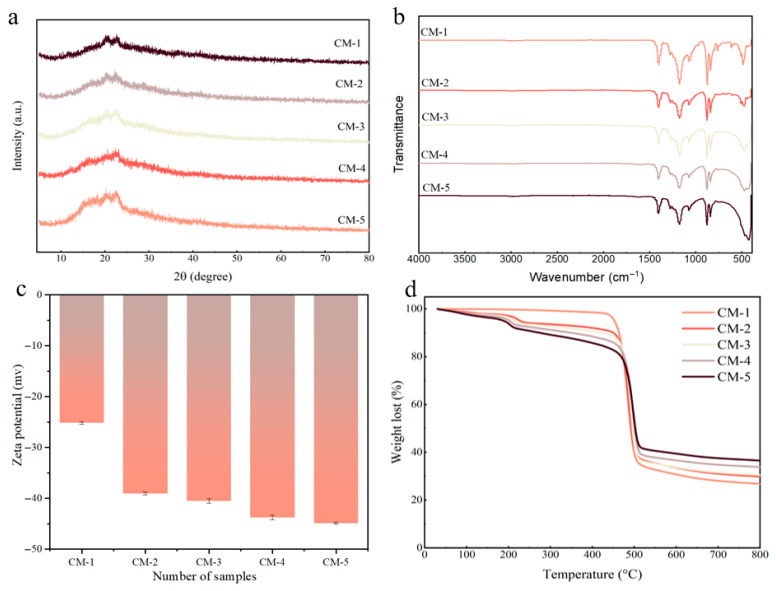
(**a**) XRD, (**b**) FT-IR, (**c**) zeta potential, and (**d**) TG of CM-1, CM-2, CM-3, CM-4, and CM-5.

XRD measurements were conducted to obtain structural information about the composite membranes. As presented in Figure 4a, the diffraction peaks at 2θ = 14.1°, 16.9°, and 20.5° were attributed to the α (100), α (020), and combined β [(110), (200)] crystal planes of PVDF, respectively [20,21], which appeared in all the membranes. Following the incorporation of GO, a distinct characteristic diffraction peaks of GO was observed at 2θ = 12°, confirming the successful introduction of GO into the PVDF matrix. Furthermore, no obvious characteristic diffraction peaks of Cu-MnO_2_ could be detected in the XRD spectrum. This phenomenon can be ascribed to two primary factors: the relatively low loading content of Cu-MnO_2_ and the strong diffraction intensity of PVDF.

FT-IR spectroscopy was employed to analyze the chemical structures of catalytic membranes. As shown in Figure 4b, the characteristic peaks of the PVDF matrix were typically observed in the range of 1000–1500 cm^−1^, and these peaks were present in all FT-IR spectra, confirming that PVDF acted as the basic framework for all membranes [22]. Due to the amount of GO added being relatively small, no obvious absorption peaks related to it were observed [23,24]. In contrast, following the incorporation of Cu-MnO_2_, distinct characteristic peaks related to Cu-O and Mn-O stretching vibrations appeared obviously in the range of 400–500 cm^−1^ [25,26,27]. Notably, with the Cu-MnO_2_ loading increasing from CM-3 to CM-5, the intensity of these peaks gradually enhanced, indicating that more Cu-MnO_2_ was successfully loaded into the membrane matrix.

As shown in Figure 4c, the zeta potentials of the catalytic membranes were characterized. For CM-1, the zeta potential was about −25 mV, and that became more negative following the incorporation of GO (CM-2), which was attributed to the abundant carboxyl and hydroxyl groups on the surface of GO nanosheets. With the introduction and gradual increase in Cu-MnO_2_ loading (CM-3 to CM-5), the zeta potential further shifted negatively, and this trend arose from the additional negative charges donated by the hydroxyl groups on the surface of Cu-MnO_2_ catalysts. As the Cu-MnO_2_ loading amount increased, more negatively charged groups were exposed on the membrane surface, thereby enhancing the membrane’s negative charge property. Collectively, the surface zeta potential of the membrane was synergistically modulated by GO and Cu-MnO_2_, and the enhanced negative charge of the membrane surface would be beneficial for promoting the membrane’s separation performance.

Figure 4d shows the thermogravimetric diagram of all membranes. A distinct mass loss was observed for CM-2, CM-3, CM-4, and CM-5 at approximately 213 °C, which was attributed to the thermal decomposition of the GO molecular chains. All membranes retained substantial thermal stability up to 500 °C without additional weight loss, while beyond this temperature, their mass began to decrease, and the trend was consistent with the typical thermal degradation behaviors of GO and PVDF matrices. Above 500 °C, CM-1 exhibited more pronounced mass loss compared to CM-5, which was attributed to the thermal shielding effect of the retained Cu-MnO_2_. These results demonstrate that the incorporation of Cu-MnO_2_ significantly improved the thermal stability of the membranes.

**Figure 5:**
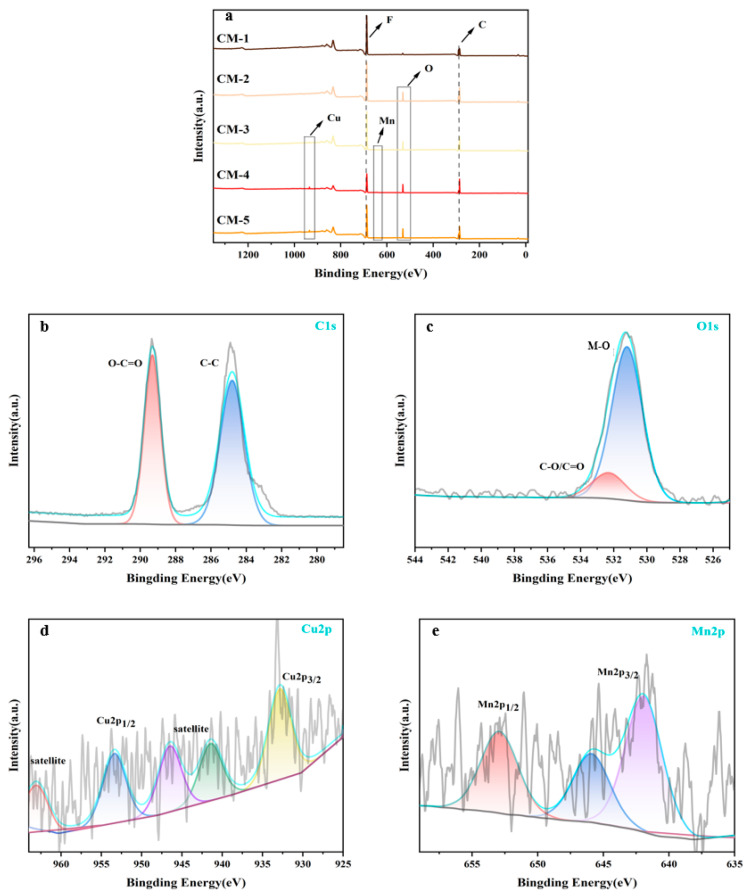
XPS spectra of membranes: (**a**) five membranes; (**b**) C1s on CM-5; (**c**) O1s on CM-5; (**d**) Cu2p on CM-5; (**e**) Mn2p on CM-5.

The surface chemical composition of the Cu-MnO_2_/GO/PVDF catalytic membrane was further investigated by XPS. In Figure 5a, the XPS wide-scan survey spectrum confirmed that only the C and F elements were present in CM-1, with their characteristic peaks appearing at 285.84 eV and 687.66 eV, respectively. In contrast, the additional O element appeared at 531.72 eV in the spectra of CM-2. Furthermore, the characteristic peaks corresponding the O, Mn, and Cu elements were identified at 79.77 eV, 687.95 eV, and 769.28 eV in the spectra of CM-3, CM-4, and CM-5. These results were consistent with the designed chemical composition of the membranes. High-resolution XPS spectra of CM-5 were further analyzed to clarify the chemical states of key elements. For the C1s spectrum, two prominent peaks were deconvoluted at 284.8 eV (C-C) and 287.8 eV (O-C=O). The C-C peak corresponded to the carbon skeletons of both PVDF and GO, while the O-C=O peak was attributed to the carboxyl groups of GO. In the O1s spectrum, two deconvoluted peaks were observed at 531.8 eV (M-O) and 533.2 eV (C-O/C=O), which were characteristic of oxygen in Cu-MnO_2_ and oxygen-containing functional groups (carboxyl and hydroxyl) from GO, respectively. The Cu2p spectrum exhibited two main peaks at 934.6 eV (Cu2p_3_/_2_) and 954.4 eV (Cu2p_1_/_2_), accompanied by satellite peaks, indicating the coexistence of Cu^2+^ (dominant) and Cu^+^ in the membrane. The presence of Cu^+^ was beneficial for the Fenton-like catalytic activity, as it can efficiently activate oxidants to generate reactive oxygen species. For the Mn2p spectrum, the peaks at 641.8 eV (Mn2p_3_/_2_) and 653.6 eV (Mn2p_1_/_2_) suggested the presence of MnO_2_, while the peak at 643.2 eV corresponded to Mn^3+^. The coexistence of Mn^4+^/Mn^3+^ redox pairs would provide abundant active sites for oxidant activation and synergize with Cu^2+^/Cu^+^ pairs to enhance the Fenton-like catalytic performance of the composite membranes.

### 3.2. Membrane Performance

#### 3.2.1. Membrane Flux and Interception

**Figure 6:**
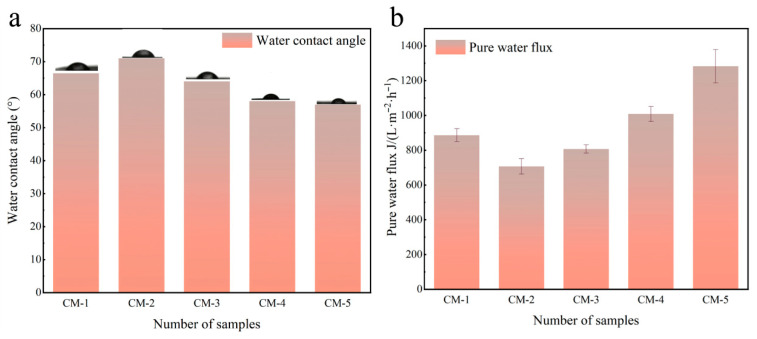
(**a**) Water contact angle of CM-1, CM-2, CM-3, CM-4, and CM-5. (**b**) Pure water flux and water contact angle of CM-1, CM-2, CM-3, CM-4, and CM-5.

The contact angle is an important parameter for studying the hydrophilicity of the membrane surfaces. Generally, a smaller contact angle indicates superior hydrophilicity of the membrane [28]. The water contact angles of the membranes were measured and the results are shown in Figure 6a. The CA of CM-1 was 66.5°, which was attributed to the inherent hydrophobicity of PVDF. In contrast, the CA of CM-2 increased to 74°, and this increase was due to the formation of a monolayer GO coating [29], as the incorporation of GO resulted in a denser surface structure compared to CM-1. With the increase in Cu-MnO_2_ content, the CA of the membranes decreased from 74° to 57°, indicating a significant improvement in membrane hydrophilicity, which led to a significant increase in the water flux of the catalytic membrane. However, the CA did not continue to decrease with the further increase in Cu-MnO_2_ loading. As shown in Figure 6b, the pure water flux of CM-2 was only 671.61 L·m^−2^·h^−1^. After the incorporation of Cu-MnO_2_, the flux of the membrane increased significantly. This is because the insertion of Cu-MnO_2_ into the GO nanosheets enlarged the interlayer spacing of GO. The synergistic effect between these components established efficient water transport channels, facilitating the unimpeded passage of water molecules. Consequently, with the increasing content of Cu-MnO_2_ in the catalytic membranes, the water flux increased gradually, for which the highest pure water flux of CM-5 reached 1391.20 L·m^−2^·h^−1^.

**Figure 7:**
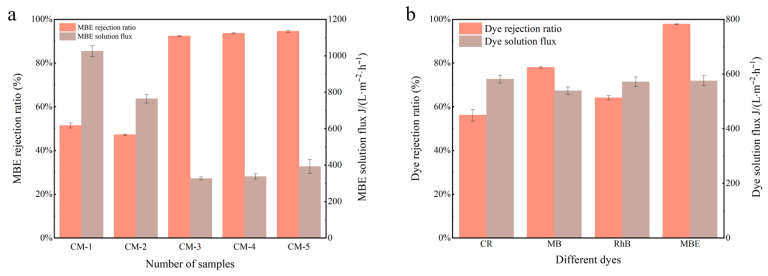
(**a**) The flux and rejection ratio of all membranes for MBE solution (C_o_ = 80 mg/L); (**b**) the flux and rejection ratio of CM-5 for CR, MB, Rh B, and MBE solution (C_o_ = 40 mg/L).

The separation performance of all prepared membranes was tested using 80 mg/L methyl blue (MBE) dye. As illustrated in Figure 7a, the dye rejection rate of the CM-1 membrane was as low as 50.21%. With the incorporation of GO, the membrane surface structure became denser compared to CM-1, so the dye flux and rejection rate of the CM-2 membrane decreased. Following the incorporation of Cu-MnO_2_, the dye rejection performance was significantly enhanced, with CM-5 achieving an MBE rejection rate of 95.10%. This improvement was attributed to the synergistic effect of physical sieving and the Fenton-like degradation during the membrane separation process after Cu-MnO_2_ loading. This combination of excellent dye degradation efficiency and sustained water permeation capability justified the selection of CM-5 as the representative catalytic membrane for subsequent experiments.

As illustrated in Figure 7b, various simulated dye wastewaters were prepared under neutral conditions to evaluate the separation performance of CM-5. The rejection rates of CM-5 for CR (negatively charged), MB (positively charged), Rh B (positively charged), and MBE (negatively charged) were measured at 58.27%, 78.25%, 64.77%, and 98.06%, respectively. Notably, the membrane did not exhibit obvious pH selectivity. Instead, the separation performance was primarily dependent on the molecular weight of the dye molecules. Among the tested dyes, the one with the largest molecular weight (MBE) exhibited the highest rejection rate, consistent with the size-sieving effect of the membrane. To further investigate the degradation performance toward macromolecular dyes, MBE was selected as a model pollutant for subsequent evaluation of the separation capability of M-5.

**Figure 8:**
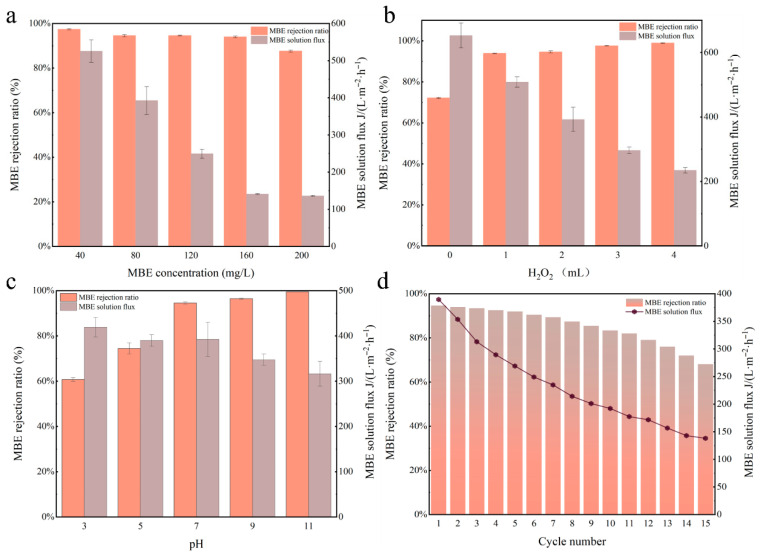
The flux and rejection ratio of MBE with different concentrations (**a**), different dosage of H_2_O_2_ (**b**), different pH values (**c**), and the reusability (**d**).

The effect of dye solution concentration on membrane performance is shown in Figure 8a. As the dye concentration increased from 40 mg/L to 200 mg/L, the rejection rate remained nearly constant at approximately 88.15%, with no significant reduction observed. However, the flux of the dye solution decreased noticeably with increasing concentration. This flux reduction was attributed to the higher density of dye molecules in the solution and their adsorption onto the membrane surface, coupled with the steric hindrance caused by their large molecular size, which hindered water permeation. At the dye concentration of 200 mg/L, the flux was recorded as 137.53 L·m^−2^·h^−1^.

As shown in Figure 8b, the rejection rate of CM-5 for an 80 mg/L MBE solution was 71.94% in the absence of H_2_O_2_. After the addition of 1 mL of H_2_O_2_, the rejection rate rapidly increased to 93.99%, and further rose to 99.06% with the addition of 4 mL H_2_O_2_. In the absence of H_2_O_2_, MBE separation by the CM-5 was achieved solely through the physical sieving effect of its porous structure. Upon the introduction of H_2_O_2_, in addition to physical interception, a heterogeneous Fenton-like reaction was triggered by the synergistic effect between Cu-MnO_2_ and H_2_O_2_, enabling a higher separation performance for MBE wastewater. However, when excessive H_2_O_2_ was added, no substantial improvement in rejection rate was observed, which was attributed to the saturation of reactive oxygen species in the system.

The effect of different pH values on the separation efficiency is presented in Figure 8c. At pH = 11, the rejection efficiency of CM-5 for MBE reached 100%, and it remained as high as 96.70% at pH = 9. Under acidic conditions (pH = 5 and pH = 3), although the rejection efficiency decreased compared to alkaline conditions, it was still maintained at a high level. For Fenton-like reaction, high catalytic activity was typically observed under acidic conditions, whereas catalytic reactivity became very limited in alkaline environments [30]. However, after incorporating the catalyst into the catalytic membrane, the pH applicability was significantly expanded.

To evaluate the long-term stability of the membrane, cyclic reuse tests were conducted. Figure 8d shows the rejection efficiency and flux of CM-5 during 15 cycles using an 80 mg/L MBE solution. As the number of cycles increased, the water flux gradually decreased, which was likely due to partial clogging of membrane pores by adsorbed dye molecules and accumulated reaction byproducts. However, the rejection ratio remained above 90% up to the 10th cycle. Nevertheless, after 15 cycles, the rejection efficiency of CM-5 decreased to 68.13%, but no visible physical damage or structural deformation of the membrane was observed. These results demonstrate that the CM-5 exhibited satisfactory cyclic stability. In addition, ICP-OES was employed to quantify the leaching concentrations of Cu and Mn ions in the treated wastewater after the 14th reuse cycle of the catalytic membrane. The detected concentrations of Cu and Mn were 0.0087 mg/L and 0.069 mg/L, respectively. Both values are below the limits specified in China’s national standard for drinking water quality (GB5749-2022 ) [31], which stipulates maximum allowable concentrations of 1 mg/L for Cu and 0.1 mg/L for Mn.

Furthermore, a comparison of the separation performance of CM-5 with that of catalytic membranes documented in existing studies is presented in Table 2. Notably, the Cu-MnO_2_/GO/PVDF catalytic membranes fabricated in this work exhibited high dye flux and superior rejection ratio, which demonstrated significant performance advantages over previously documented membranes.

#### 3.2.2. Study on the Stability of the Membrane

**Figure 9:**
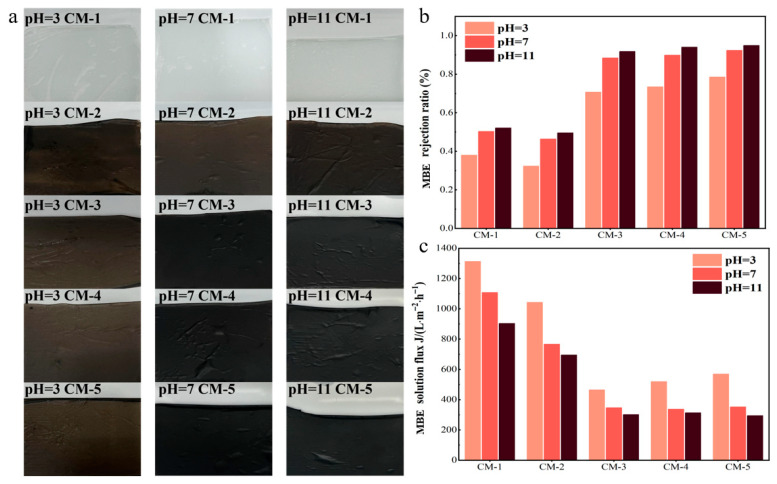
(**a**) Photos of catalytic membrane after immersion in solutions with different pH values for 30 days; the MBE rejection ratio (**b**) and flux (**c**) of catalytic membrane after immersion in solutions with different pH values.

To evaluate the structure stability of the membranes, they were immersed in aqueous solutions with different pH values (pH = 3, 7, 11) for 30 consecutive days. As shown in Figure 9a, no visible damage, structural deformation, or peeling was observed on any of the membranes after the immersion period. Subsequently, the rejection rate (Figure 9b) and flux (Figure 9c) of CM-5 for an 80 mg/L MBE solution were measured. For CM-1 and CM-2, both the rejection ratio and MBE flux decreased obviously after immersion, indicating the poor chemical stability of pure PVDF membrane and GO-PVDF composite membrane. In contrast, CM-3, CM-4, and CM-5 exhibited no significant fluctuations in MBE rejection and flux following the 30 days of immersion. These results demonstrate that the incorporation of Cu-MnO_2_ markedly increased the stability of the catalytic membranes.

#### 3.2.3. Degradation Mechanism

**Figure 10:**
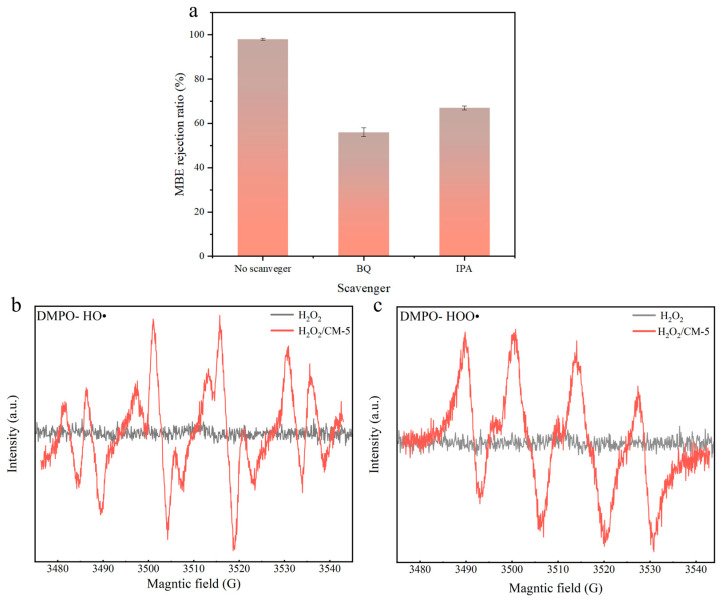
(**a**) Free radical scavenger experiment; (**b**) EPR signals of DMPO- HO· adducts; (**c**) EPR signals of DMPO- HOO· adducts.

To investigate the role of free radicals in the filtration process, isopropanol (IPA, a HO· scavenger) and benzoquinone (BQ, a HOO· scavenger) were separately added to the MBE solution. As illustrated in Figure 10a, the addition of IPA reduced the MBE rejection rate from 98.6% to 67.3%, while a more significant decline (from 98.8% to 56.5%) was observed upon BQ introduction. These results confirm that both H O· and HOO· radicals played crucial roles in the catalytic filtration mechanism. To further verify the generation of HO· and HOO· in the Fenton-like system, EPR characterization was performed and the results are presented in Figure 10. As shown in Figure 10b, distinct DMPO-HO· (1:2:2:1) adduct signals and DMPO-HOO· (1:1:1:1:1) adduct signals were detected in the reaction system containing CM-5 and H_2_O_2_. In contrast, no corresponding adduct signals were observed in the EPR spectrum of the system without CM-5. These detection results indicate that the Fenton-like oxidation reaction occurred during the membrane separation process.

**Figure 11:**
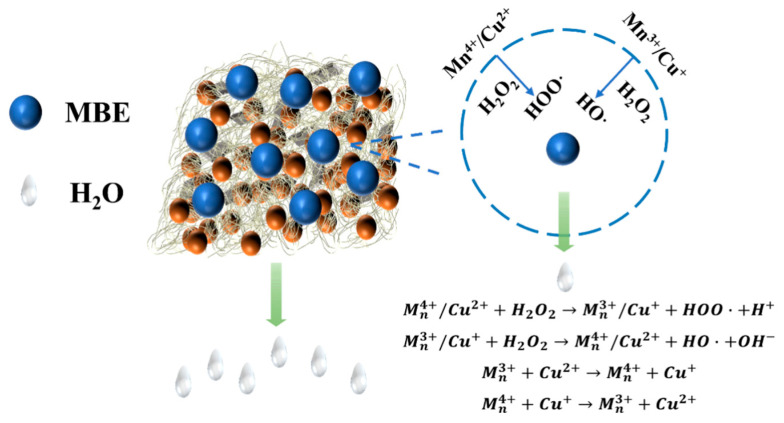
The filtration mechanism of MBE by Cu-MnO_2_/GO/PVDF catalytic membrane.

Based on the above results, the filtration mechanism of the Cu-MnO_2_/GO/PVDF catalytic membrane for MBE removal is illustrated in Figure 11. The membrane, integrated with Cu-Mn active sites, triggered a series of redox reactions with H_2_O_2_. Specifically, HO· and HOO· were generated during the reaction between metal ions and H_2_O_2_, and these reactive oxygen species acted as strong oxidants to efficiently degrade MBE. Meanwhile, the redox cycles of Mn (Mn^4+^-Mn^3+^) and Cu (Cu^2+^-Cu^+^) ensured the continuous regeneration of active sites, maintaining long-term catalytic activity. During this process, water molecules passed through the membrane’s efficient transport channels, while MBE molecules were either degraded by the reactive radicals or physically retained by the membrane’s porous structure, thus achieving effective filtration and catalytic degradation simultaneously.

## 4. Conclusions

In this study, a Cu-MnO_2_/GO/PVDF catalytic membrane was successfully fabricated via a phase inversion method. The incorporation of Cu-MnO_2_ into the GO interlayers effectively tailored the membrane’s porous structure and constructed efficient transport channels for water molecules. The resulting membranes exhibited remarkable comprehensive performance; notably, the optimal membrane CM-5 maintained exceptional separation efficiency across a wide pH range and demonstrated excellent reusability and long-term stability. Mechanistic studies revealed that the superior performance of Cu-MnO_2_/GO/PVDF membranes originated from the synergistic effect of physical sieving and heterogeneous Fenton-like catalytic degradation. Thus, this work provides a novel and scalable strategy for the design and fabrication of multifunctional catalytic membranes, and the developed Cu-MnO_2_/GO/PVDF membranes hold great potential for practical application in the treatment of complex dye-containing wastewater.

## Figures and Tables

**Table 1:** Separation membranes with different components.

Type of Membrane	GO (g)	Cu-MnO_2_ (g)	PVDF (g)	NMP (mL)
CM-1	0	0	3	30
CM-2	0.5	0	3	30
CM-3	0.5	0.2	3	30
CM-4	0.5	0.5	3	30
CM-5	0.5	0.8	3	30

**Table 2:** Comparison the performance of catalytic membranes in former studies.

Substrate	Catalyst	Dye	Co (PPm)	J_dye_ (L m^−2^ h^−1^)	Testing Condition (MPa)	R_dye_ (%)	Ref
PVDF	PVP-MnO_2_	BPA	10	469.02	0.1	100	[22]
PVDF/GO	NH_2_-MIL-88B (Fe)	MB	20	335.07	0.1	99.6	[32]
CS/GO/PVDF	α-MnO_2_	MBE	40	923.72	0.1	97.78	[33]
PVDF	NC/TiO	MB	1	274.23	0.1	98	[34]
PVDF	CuO-Fe3C-C	TC	12	422.27	0.1	97.9	[35]
PVDF	SMA/CuS	RhB	10	735.3	0.1	99.7	[36]
PVDF	MgFe_2_O	RhB	20	1792	0.1	99	[37]
PAN-Si	Ag NPs	Methyl orange	1	230	0.1	99	[38]
PVDF	Cu-MnO_2_-GO	MBE	80	434.94	0.1	100	This work

## Data Availability

The original contributions presented in this study are included within this article; further inquiries can be directed to the corresponding author.
